# Bilateral Idiopathic Sclerochoroidal Calcifications

**DOI:** 10.2174/1874364101711010076

**Published:** 2017-04-27

**Authors:** Venkatesh L. Brahma, Sumit P. Shah, Nauman A. Chaudhry, Jonathan L. Prenner

**Affiliations:** 1 NJ Retina, Robert Wood Johnson Medical School, Rutgers RWJ – University Hospital, New Brunswick, NJ, USA; 2 New England Retina Associates, PC, New London, CT, New York, USA

**Keywords:** Bilateral calcifications, Idiopathic calcifications, Sclerochoroidal calcifications, Bartter’s syndrome, Gitelman’s syndrome

## Abstract

**Background:**

Sclerochoroidal calcification (SCC) is a rare and benign condition found mostly in middle-aged and elderly Caucasian men, characterized by multiple yellow-white lesions seen most commonly in the temporal regions of the fundus. While they may be concerning for benign tumors, primary neoplasias or metastases, SCCs most commonly present as asymptomatic findings during routine ophthalmologic testing and have a very good prognosis as they rarely cause visual deficits.

**Objective:**

To report and describe the findings in a case of bilateral idiopathic sclerochoroidal calcifications.

**Methods:**

A retrospective case report.

**Results:**

Repeated ophthalmological exams, including fundoscopic examination, ultrasonography, optical coherence tomography and fluorescein angiography, were all consistent bilateral idiopathic sclerochoroidal calcifications.

**Conclusion:**

While most cases of idiopathic sclerochoroidal calcifications represent a benign ophthalmological condition, there are known associations with other systemic conditions, such as hyperthyroidism, hyperparathyroidism, Bartter’s syndrome and Gitelman’s syndrome. It is for this reason that these patients warrant a full systemic work-up in addition to careful ophthalmological monitoring.

## CASE REPORT

 A 71-year old white male was referred for hyperopic refractive shift. On exam, best corrected visual acuity was 20/25 in both eyes. Anterior segment exam was unremarkable. Fundus examination revealed multiple (around 2-3) yellowish elevated choroidal lesions in both eyes, the largest one with a diameter of approximately 10 mm. seen throughout the superotemporal macula as well as the superotemporal periphery. There was no associated subretinal fluid, heme or significant retinal pigment epithelium changes. B-scan ultrasonography showed moderate solid appearing elevations (<4mm), as well as high internal reflectivity. Systemic work up for potential associations was negative; the patient underwent extensive systemic workup including a complete blood count, comprehensive metabolic panel (including serum calcium testing), phosphorus and magnesium levels, all of which were within normal limits.

**Fig. (1):**
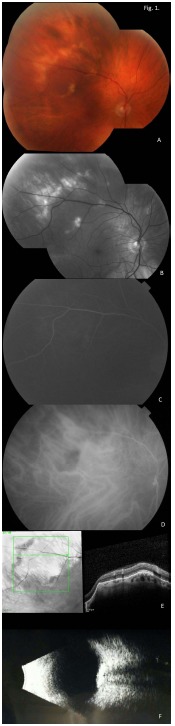
(Right Eye Images) **A.** Montage fundus photographs show yellow-white lesions in the superotemporal macula and periphery of the right and left eyes. **B**. Red-Free montage images highlight the lesions. **C.** Fluorescein angiogram shows staining. **D.** ICGA showed hyper-fluorescence in the corresponding region. **E.** OCT images show an elevated lesion of the choroid. **F.** B-scan ultrasound show solid appearing hyper-echoic elevations in the sclera and choroid with posterior shadowing.

**Fig. (2):**
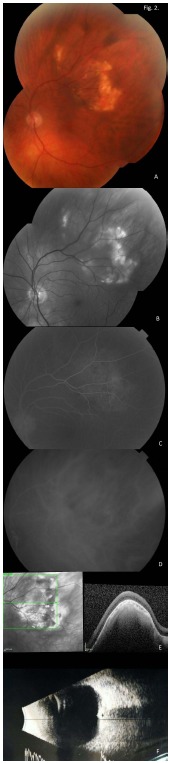
Respective left eye images.

Montage fundus photographs show yellow-white lesions in the superotemporal macula and periphery of the right and left eyes (Figs. **1A** and **2A**, respectively). Red-Free montage images highlight the lesions in the right and left eyes (Figs. **1B** and **2B**, respectively). Fluorescein angiogram shows staining in the late phase of the right and left eyes (Figs. **1C** and **2C**, respectively). Indocyanine green angiography showed hyper-fluorescence in the corresponding regions of the right and left eyes (Figs. **1D** and **2D**, respectively). Optical coherence tomography images show an elevated lesion of the choroid in the right and left eyes (Figs. **1E** and **2E**, respectively), with maintenance of the normal retinal architecture. Lastly, B-scan ultrasonography showed solid appearing hyper-echoic elevations in the sclera and choroid with posterior shadowing in the right and left eyes (Figs. **1F** and **2F**, respectively).

## CONCLUSION

Sclerochoroidal calcifications warrant a full ophthalmologic and systemic workup as a minority of the cases of SCCs are associated with endocrinologic pathology including hyperthyroidism and hyperparathyroidism [1]. Associations have also been reported with nephrologic pathology such as Bartter’s [2] and Gitelman’s syndromes [3]. Therefore, thyroid hormone levels, calcium and phosphorus levels, magnesium levels, and BUN and creatinine are all recommended for patients suspected to have sclerochoroidal calcifications. Additionally, these patients should undergo routine ophthalmological evaluation to assess for any further progress of the disease process.

## LIST OF ABBREVIATION

SCC: = Sclerochoroidal Calcification
